# Isolation, Characterization, and Effect on Biofilm Formation of Bacteriocin Produced by *Lactococcus lactis* F01 Isolated from *Cyprinus carpio* and Application for Biopreservation of Fish Sausage

**DOI:** 10.1155/2022/8437926

**Published:** 2022-11-22

**Authors:** Ulrich Daquain Fotso Techeu, Pierre Marie Kaktcham, Hector Kenfack Momo, Edith Marius Foko Kouam, Laverdure Tchamani Piame, Romial Joel Ngouenam, François Zambou Ngoufack

**Affiliations:** ^1^Research Unit of Biochemistry of Medicinal Plant, Food Science and Nutrition (URBPMAN), Department of Biochemistry, Faculty of Science, University of Dschang, P.O. Box 67, Dschang, Cameroon; ^2^Faculty of Medicine and Pharmaceutical Science, P.O. Box 67, Dschang, Cameroon

## Abstract

The aim of this work was the screening of bacteriocin-producing LABs isolated from fish, the selection of promising/prominent strain(s), the characterization of the bacteriocin produced, and the evaluation of its potential to be used as biopreservative(s). Amplification and sequencing of the 16S rRNA gene of the bacteriocin-producing strain was performed. Then a partial purification of the produced bacteriocin, using a combination of ammonium sulfate and chloroform-methanol precipitation, was done. Its molecular weight was determined by SDS-PAGE. In addition, the action spectrum, the hemolysis test, and its ability to inhibit biofilm formation were analyzed. A total of 88 isolates of lactic acid bacteria (LAB) including one bacteriocin producer, which was identified as *Lactococcus lactis* F01, were collected. The bacteriocin was partially purified with an estimated yield of 40%. Regarding the SDS-PAGE profile, the secreted bacteriocin has molecular weight of about 3.5 kDa and was identified as class I bacteriocin. The antimicrobial test showed that the bacteriocin inhibits pathogenic and/or spoilage bacteria, 10 Gram-positive and 16 Gram-negative bacterial species. Moreover, it can inhibit biofilm formation from 1.3% (*Escherichia coli*) to 63.92% (*Pseudomonas aeruginosa* ATCC15692) depending on the strain. The hemolytic activity of novel bacteriocin was observed at the concentration of 10 *μ*g/ml of bacteriocin crude extract, which was 0.7 ± 0.0029%. In addition, it exhibited good thermal and pH stability with retained antibacterial activity of 85.25% after treatment at 121°C for 20 min, as well as at a pH range between 2.0 and 10.0. Moreover, this bacteriocin showed the ability to inhibit the growth of bacterial culture load in fish sausage stored at 8°C for 28 days. Considering the results obtained, bacteriocin could be potentially exploited as an alternative to chemical preservatives or as a substitute for antibiotics.

## 1. Introduction

The quality of human being life has been significantly improved due to continuous technological development through the introduction of new substances capable of improving food preservation and ensuring its safety [1]. However, most of these preservatives used in the food industry are chemicals containing organic acids and salts as main components. However, some studies have highlighted the harmful effect of these preservatives on the health of consumers [2]. These chemicals can lead to several diseases such as colon cancer, which are more observed in recent decades and related to the side effects caused by the abuse or overuse of chemical preservatives (E249 to E252) according to some studies [3–5]. So, there is an urgent need to develop a new natural, effective, and safe preservative as an alternative to these harmful ones [1, 6]. In this perspective, work has been carried out on the bacteriocins of lactic bacteria isolated from milk and fruits to understand their structure-function relationships and their mode of action and to promote their applications in the fight against pathogens, in particular multidrug resistant (MDR) [5, 7–10]. Indeed, some studies have reported that bacteriocins such as nisin, mersacidin, or lacticin can be considered as an alternative to control the proliferation of pathogenic bacteria in foods [11] and even those that are resistant to antibiotics [12]. In the same idea, the work of [13–15] focused on the inhibitory power of bacteriocins against spoilage bacteria and many pathogenic bacteria (MR). Thus, bacteriocins can be used as an alternative to chemical preservatives on the one hand and on the other as an alternative to antibiotics, as has been the subject of many studies for decades [16]. Despite the discovery of several bacteriocins and their use as alternatives to chemical additives, only nisin is accepted and approved by FDA so far [17]. The limitations in the application of certain bacteriocins have made it as a hot spot for the discovery of new research to improve known bacteriocins [18]. Considering these limitations, there is a need to search for a new bacteriocin-producing strains. Different niches can be explored such as natural fermented products, the gastrointestinal tracts of animals, and fish as a source of isolation [7]. To the best of our knowledge, some works such as those of [9, 19] which focused on the quantitative analysis of the bacterial microbiota of farmed fish, tilapia, and carp and on the inhibitory activity of these bacteriocin-producing strains against increase in the number of pathogenic bacteria of fish. Then, based on the in vitro evaluation/assessment of the probiotic properties of bacteriocinogenic and nonbacteriocinogenic lactic acid, bacteria from the intestines of Nile tilapia and common carp conclusions were drawn for their use as probiotics in aquaculture. Having in mind the previously obtained results, we set the goal to characterize the bacteriocin produced by a LAB strain isolated from “*Cyprinus carpio*” and to apply it in the biopreservation of fish sausages with the aim of possible use as a food biopreservative.

## 2. Materials and Methods

### 2.1. Bacterial Strains, Media, and Growth Conditions

Seven strains and 15 pathogenic isolates (used as indicator organisms in this study) were obtained from the Research Unit of Biochemistry of Medicinal Plants, Food Science, and Nutrition (URBMAN), of the University of Dschang, which were isolated during the microbiological analysis of laboratory of the District Hospital of Dschang. The strains and the indicator isolates included in this work were *Bacillus subtilis* ATCC14453, *Staphylococcus aureus*, *Escherichia coli*, *Klebsiella pneumonia* ATCC 35595, *Pseudomonas aeruginosa* ATCC 15692, *Salmonella* Typhimurium ATCC 14028, *Bacillus amyloliquefaciens*, *Clostridium* sp., *Escherichia* sp. 3B, *Serratia* sp., *Yersinia* sp., *Salmonella* sp. 3B, *Vibrio* sp. 2DS, *Vibrio* sp. 1DS, *Salmonella* sp. 2B, *Pseudomonas* sp. Z, *Staphylococcus* sp. 2B, *Staphylococcu*s sp. 1B, *Pseudomonas* sp., *Shigella* sp., *Escherichia* sp. 2B, and *Staphylococcus* sp. 1B. All pathogenic bacteria were reactivated in BHI (Lab a, Neogen Company, United Kingdom) at 37°C for 24 h, and the bacteriocin-producing LAB strain was grown in MRS broth medium (Titan Biotech LTD, Indian) at 37°C for 18 h.

### 2.2. Isolation, Screening, and Identification of Bacteriocinogenic Strains

#### 2.2.1. Isolation of the Strains

Samples of fish and fish products were collected from fish farmers (*Cyprinus carpio* and *Arius africanus*) in the town of Bâtie 5° 18′ 53^″^ North, 10° 19′ 31^″^ East (West Cameroon) and from fishermen (*Sardina pilchardus*, *Silurus glanis*, *Penaeus monodon*, *Oreochromis niloticus*, and *Penaeus indicus*) in the area of Youpwe 4° 00-4° 8′ North, 9° 30′-9° 49^″^ East (Littoral region of Cameroon, from November 2020 to January 2021) and brought on ice to the laboratory for analysis. One gram of the intestinal contents of each sample was mixed in 10 ml of sterile normal saline (0.9% *w*/*v* NaCl). Then, this mixture was serially diluted six times; 0.1 ml of the dilutions of the samples was plated on MRS agar plates (Titan Biotech LTD, Indian) and incubated anaerobically at 37°C for 24-48 h. Then, phenotypic and genotypic identification was performed.

#### 2.2.2. Detection of Antagonistic Activity

Antimicrobial activity of selected isolates was tested by the spot on the lawn agar diffusion method as described previously by [8] with some modifications. The inoculum of tested bacterial isolates of 2% was added to fresh MRS broth and incubated for 18 h at 37°C. After incubation, the cultures were centrifuged at 10,000 g for 10 min at 4°C and the supernatant was recovered and neutralized with 1 M NaOH solution to pH 6.5. Each sterile Petri dish was poured with 10 ml of semisolid BHI (0.7% agar) and overlaid with MRS semisolid medium, which was previously inoculated with 2% of overnight culture of the indicator strains. After solidification, 10 *μ*l of the neutralized cell-free supernatant (NCFS) was spotted on the surface of the inoculated medium, left to dry, and then incubated at 37°C for 24 h. The determination of bacteriocin activity in arbitrary units (AU) was performed as follows: serial double dilutions of NCFS were made in sterile distilled water. Petri dishes containing the indicator strains were prepared as described above, and 10 *μ*l of each dilution of NCFS was spotted on each dish and incubated at 37°C for 24 h. After that, the plates were examined for the appearance of clear zones, and the bacteriocin titer was defined according to the following formula:

(1) $$\begin{array}{c} T=\frac{\left(100\times D\right)}{\left(V\right)}, \end{array}$$

where *T* is the bacteriocin titer in AU/ml, *V* is the volume of the spotted supernatant (10 *μ*l), and *D* is the dilution factor.

#### 2.2.3. Identification of the Bacteriocinogenic and Susceptibility Strains

Phenotypic and physiological properties of the bacterial isolates were tested (arginine hydrolysis, carbohydrate fermentation, and growth capacity in MRS medium supplemented with 4.5, 6.5, and 9.6% NaCl, respectively). Molecular identification of isolates was performed by amplifying the 16S rRNA gene by PCR using the universal primers 27F (5′-AGAGTTTGATCCTGGCTCAG-3′) and 1492R (5′ GGTTACCTTGTTACGACT-3′) [20]. The 16S rRNA gene was sequenced, and the sequence was compared with the NCBI GenBank database to perform homologous analysis using BLAST search [21] (http://blast.ncbi.nlm.nih.gov/Blast.cgi).

### 2.3. Characterization of the Bacteriocin Produced by *Lactococcus lactis* F01

#### 2.3.1. Growth and Production Kinetics of Bacteriocin

Test of growth-dependent production of bacteriocin was performed according to the method described by [22, 23]. One hundred milliliters of MRS broth was inoculated with 2% of the previously reactivated overnight culture and was incubated at 37°C for 18 h. Aliquots of 6 ml were taken every 2 h and subdivided into 2 parts. The first parts (2 ml) were used for optical density reading (600 nm), and the remaining (4 ml) were used for bacteriocin titer (AU) determination as described previously.

#### 2.3.2. Partial Purification and Determination of Bacteriocin Molecular Weight

NCFS was subjected to ammonium sulfate (70% PSA) precipitation, and the precipitate was collected by centrifugation (10,000 g for 10 min at 4°C) and dissolved in sterile phosphate buffer (20 mM, pH 6.5). The resulting precipitate was reprecipitated using chloroform/methanol (PCM) in proportions (2 : 1 *v*/*v*) as described by [24]; the bacteriocin titer at each purification step was calculated as described previously (paragraph 2-3). In addition, the protein concentration of each fraction was determined by the Bradford method [25]. The determination of the molecular weight and the number of bacteriocins produced by the LAB strain was performed by Tris-glycine SDS-PAGE [26]. Molecular weight markers used were BSA (66 kDa) and nisin (3.5 kDa) prepared at the concentration of 1 mg/ml.

#### 2.3.3. Action Spectrum of Bacteriocin

NCFS and semipurified bacteriocins were tested for their inhibitory activities against pathogenic strains and 22 isolates by the spot on the lawn diffusion method on agar medium as described previously.

#### 2.3.4. Mode of Action of Bacteriocin

The effect of bacteriocin on pathogenic bacteria *Salmonella* Typhimurium ATCC 14028, *Pseudomonas aeruginosa* ATCC 15692, and *Klebsiella pneumonia* 35595 was performed according to the method of [27, 28]. An aliquot (0.2 ml) of the partially purified bacteriocin was added to 10 ml of an indicator strain at the beginning of the exponential phase (4 h), and the mode of action was monitored by reading the optical density (OD) at 600 nm of the pathogenic strain growth during each 2 h for 18 h at 37°C. OD was compared with that obtained for the growth of the pathogenic strain without bacteriocin.

#### 2.3.5. Quantification of Biofilm Formation

The ability of strains to form biofilms was determined following the method of 20 *μ*l of a 24 h old culture of the pathogenic strains was inoculated into 180 *μ*l of sterile liquid MHB medium in wells of a 96-well microplate in triplicate. As a negative control, 200 *μ*l of MHB medium was used. After incubation at 37°C for 48 h, the wells were rinsed three times with 200 *μ*l of phosphate-buffered saline to remove unadhered cells to the wells. After drying the plates at 60°C for 1 h, 180 *μ*l of 2% crystal violet was added to the wells and stained for 15 min. Excess dye was removed, and then, the dye bound to the adhered cells were solubilized with 180 *μ*l of 33% glacial acetic acid, and OD was measured at 570 nm using a microplate spectrophotometer reader.

#### 2.3.6. Inhibition of Biofilm Formation of Pathogenic Bacteria

The method described by [29] was used to determine the biofilm formation inhibition of pathogenic bacteria by bacteriocins. For this purpose, 200 *μ*l of a culture of indicator bacteria in a liquid medium supplemented with 1% glucose was inoculated in wells of a 96-well microplate in which 50 *μ*l of partially purified bacteriocin was added. A volume of 250 *μ*l of an indicator bacterial culture was used as a positive control, and a liquid medium with 1% sterile glucose was used as a negative control. After incubation at 37°C for 48 h, the quantification of the biofilms formed was performed following the method described by [30]. From the obtained OD 570 nm, the percentages of inhibition of biofilm formation were calculated using the following formula:

(2) $$\begin{array}{c} \text{PI}=\frac{\left(\left(C‐B\right)‐\left(S‐B\right)\right)}{\left(C‐B\right)}\times 100, \end{array}$$

where *B* is the OD 570 nm of the sterile culture medium, *C* is the OD 570 nm of the culture of the indicator strains without added partially purified bacteriocin, and *S* is the OD 570 nm of the culture of the indicator strains with added partially purified bacteriocin.

### 2.4. Effect of Heat Treatment, pH, and Proteolytic Enzymes on Bacteriocin Activity

Sensitivity to heat treatment, pH, and enzymes was studied by treating 300 *μ*l of semipurified bacteriocin at different temperatures, respectively, at 100°C for 30 min, 60 min, 90 min, and 120 min (in a water bath) and in an autoclave (120°C, 1.5 bar) for 20 min. To determine pH sensitivity, 50 *μ*l of semipurified bacteriocin was adjusted to different pH ranges from 2 to 10 (range 1.0) by solubilization in specific buffer solutions (50 mM acetate buffer, pH 2.0 and 3.0; 50 mM phosphate buffer pH 4.0, 5.0, 6.0, and 7.0; and 50 mM Tris-HCl buffer, pH 8.0, 9.0, and 10.0); 10 mg/ml proteinase K (in 50 mM potassium phosphate buffer, pH 7); trypsin (in 50 mM Tris-HCl buffer, pH 8); and pepsin (in 50 mM acetate buffer, pH 2) enzyme solutions. Prepared enzyme solutions were added to NCFS to obtain a final concentration of 1 mg/ml enzyme and incubated for 2 h at 37°C. The residual activity of the different treated samples was determined by the diffusion method. Buffers with enzymes were used as negative and nontreated partially purified bacteriocin as positive control in bacteriocin assay.

#### 2.4.1. Effect of Organic Solvents, Detergents, EDTA, and NaCl

Aliquots of 400 *μ*l of semipurified bacteriocin were solubilized in 5% *v*/*v* solutions of different solvents (methanol, ethanol, chloroform, and acetate). The stock solutions of detergents (SDS, Tween 20, Tween 80, Urea, and Triton X-100), EDTA, and NaCl were prepared at the concentration of 1% (*w*/*v* or *v*/*v*) to solubilize the bacteriocin. EDTA and NaCl were added to concentrations of 5% and 7%, respectively. After homogenization, incubation was performed at 37°C for 2 h, followed by evaluation of residual activity by the diffusion method. The 5% and 7% solvent solutions were used as a negative control.

#### 2.4.2. Determination of MIC

For the determination minimum inhibitory concentration (MIC), the double microdilution media method described by ISO 10932 was used [31]. In a 96-well microtiter plate, 100 *μ*l of Mueller-Hinton broth (MHB) was added to each well. Then, 100 *μ*l of antibiotic solution or 100 *μ*l of bacteriocin was added in different wells, and successive half volume was transferred to next well (two times diluted). Finally, to each well, 100 *μ*l of bacterial suspension (adjusted to 0.5 of the McFarland scale) of each strain was added and plates were then incubated at 37°C for 18 h. The positive control was bacterial suspension without antibiotic, whereas the negative control consisted of the medium with sterile distilled water added. The MICs were compared to the critical values defined by [31].

### 2.5. Evaluation of the Hemolytic Activity of the Bacteriocin

The hemolytic activity of the bacteriocin was tested on chicken erythrocytes by the method of [32]. The erythrocytes were separated by centrifugation at 1,000 g for 5 min. The pellet was washed three times with phosphate-buffered saline (PBS 0.1 mol/l pH 7.4) and resuspended in PBS (1%). The assay consisted of mixing 50 *μ*l of the erythrocyte solution and 50 *μ*l of bacteriocin at concentrations of 86.8 *μ*g/ml, 43.4 *μ*g/m, 10 *μ*g/ml, and 2.5 *μ*g/ml or nisin at 10 *μ*g/ml. 50 *μ*l of PBS buffer added to 50 *μ*l of erythrocyte suspension was used as a negative control (NC) and, as a positive control (PC), Triton X-100 at 0.5% *v*/*v*. The assay was performed in triplicate. Samples were incubated at 37°C for 1 h with shaking and then centrifuged at 1,000 g for 5 min, and the supernatant was used to determine the amount of hemoglobin released. A volume of 50 *μ*l of the supernatant from each treatment was added, respectively, to wells of microdilution 96-well plates, and the absorbance of the samples was read at 545 nm using a microplate reader. The percent of hemolysis for each sample was calculated using the following formula:

(3) $$\begin{array}{c} \%H=\frac{\left(\text{Abs bacteria sample}‐\text{Abs CN}\right)}{\left(\text{Abs}‐\text{CP}‐\text{Abs CN}\right)}. \end{array}$$

### 2.6. Determination of Bacteriocin-Cell Wall Association of the Producer Strain

The 18 h MRS broth culture of the bacteriocin producer was centrifuged at 10,000 g for 20 min at 4°C. The antibacterial activity of NCFS was tested by the diffusion method. The cell pellet was resuspended in 95% methanol adjusted to pH 1.5 and 2 and shaken overnight at 4°C using a magnetic stirrer. The cell suspension was centrifuged at 10,000 g for 20 min at 4°C, and the supernatant was filtered through a millipore filter (0.22 *μ*m). The clear supernatant was evaporated at 65°C using a water bath for 30 min. Then, the activity of the bacteriocin was tested by the diffusion method after suspension of the pellet in ultrapure water [19], and the protein concentration was determined by the Bradford method.

### 2.7. Biopreservation Assays of the Partially Purified Bacteriocin in Fish Sausage

#### 2.7.1. Preparation of Fish Sausage Samples

Sausages were prepared using the different conditions. The following ingredients were added at the final concentration: nitrated salt: 0.2% of the total mass (flesh+skin+oil+brine). The brine was composed of celery, garlic, heads, and skin of fish, whose mixture was heated at a temperature of 80°C for 15 min; wheat flour 12.94% of flesh, skin, oil, and brine; pepper 10 g/kg of flesh; garlic 6 g/kg of flesh; and oil 25 ml/kg; and finally, the preservatives were added as follows: bacteriocin (1 ml/50 g = 243 AU/g), and a chemical preservative (sodium benzoate 1 g/kg) was added [33].

### 2.8. Microbial Analysis

ISO standards were used to determine the microbiological load of the following bacteria during 28 days: total mesophyll aerobic flora (FAMT), *Salmonella* sp., *Staphylococcus* sp., *Vibrio* sp., and *Escherichia* sp. A 25 g of slices of the preserved fish sausage was ground under aseptic conditions in a mortar with a sterile pestle and homogenized for 2 min on a vortex. Samples were serially diluted and plated in triplicate on nutrient agar (plate count agar Hi-Media) and selective agars such as thiosulfate citrate bile salts sucrose (TCBS) agar (Hi-Media), *Salmonella-Shigella* agar (SSA Hi-Media), mannitol salt agar (MSA Hi-Media), Man, Rogosa and Sharpe (MRS; Hi-Media), and *Pseudomonas* selective agar (Drigalski agar) (Hi-Media) and incubated at 37°C for 24 and 48 h for FMAT.

The total number of colonies present in the sample unit is given by the formula as follows:

(4) $$\begin{array}{c} \text{N}=\frac{\Sigma c}{\left(n1+0.1n2\right)\times d}\times \frac{1}{V}\times \frac{\text{Vsm}}{\text{Vpr}}, \end{array}$$

where *n*1 is the number of plates counted at the lowest retained dilution, *n*2 is the number of plates counted at the second retained dilution, *d* is the dilution rate at which the first counts are made: lowest dilution, ∑*C* is the total number of colonies on the retained plates, *V* is the volume of the inoculated test sample in ml, Vsm is the volume of the stock suspension in ml, and Vpr is the mass of the product (g) that made up the stock suspension.

### 2.9. Statistical Analysis

The results of the bacterial counts were presented as mean ± standard deviation using Excel version 2007 software. They were then analyzed by one-way ANOVA, and when differences were significant between means, they were compared with each other using IBM SPSS Statistics 22 software. Differences were considered significant for *p* values < 0.05.

## 3. Results and Discussion

### 3.1. Isolation and Antimicrobial Screening of Isolates

**Figure 1:**
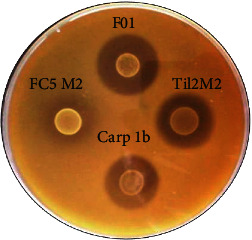
Clear area around the spots reflecting antibacterial activity against LAB.

A total of 88 Gram-positive and catalase-negative bacterial isolates were obtained from different fresh fish and fish product samples; 67 (76.13%) of these isolates were rod-shaped and thus were classified in the genus *Lactobacillus.* The remaining 21 (23.86%) isolates were with spherical cell shape and were classified in the genus *Lactococcus*. Similarly, 17 isolates of pathogenic bacteria were also isolated, among which 4 isolates of *Escherichia* sp., 3 isolates of *Staphylococcus* sp., 4 isolates of *Salmonella* sp., 3 isolates of *Vibrio* sp., and 3 isolates of *Pseudomonas* sp. Among the 88 isolates, only 12 (13.6%) showed very good antibacterial activity by inhibiting large number of indicator strains and pathogenic isolates (Figure 1). The isolate F01 showed the best antimicrobial activity (diameter of inhibition zone of 2.6 cm) on both double and triple layer media. This low percentage of bacteriocin-producing isolates from fresh fish and fish products can be justified by the composition of the isolation/selection medium and the abundance of nutrients in their environment, which may reduce the development of competitive mechanisms for their survival and adaptation. These results are in the same range as those obtained by [34]. In that study, seven LAB strains previously isolated from the gut of Nile tilapia and common carp had demonstrated potent antibacterial activity against host-derived and non-host-derived fish pathogens. According to this result, it was essential for us to identify our isolate.

### 3.2. Phenotypic and Molecular Identification of Isolate of Interest

**Figure 2:**
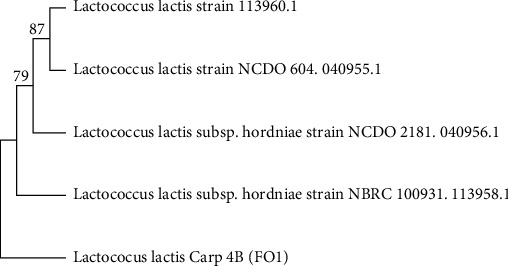
Neighbor-joining phylogenetic tree based on 16S rRNA gene sequences showing the position of strain F01.

The physiological criteria that allowed the phenotypic identification of the isolate F01 are listed in Table 1. It was found that the isolate F01 was able to grow in 4.5, but not in 6.5 and 10% of NaCl. Moreover, the strain F01 was not able to grow at alkaline pH (9.6) and to produce biogenic amines and CO_2_ in the presence of glucose. However, it was able to grow at temperatures between 8°C and 40°C. The amplified nucleotide sequence of the 16S rDNA of FO1 was a 1000 bp fragment. The 16S rDNA nucleotide sequence of FO1 showed 95.68% identity to *Lactococcus lactis* subsp. *hordniae* 113958.1. present in the NCBI GenBank database. Based on the information from the phenotypic, physiological, and molecular tests, the FO1 strain was identified as *Lactococcus lactis* F01. The 16S rRNA gene sequence of strain F01 was deposited under accession number ON000254 (Figure 2). After identification, it determined the growth and production kinetics of the bacteriocin.

### 3.3. Characterization of the Bacteriocin Produced by *Lactococcus lactis* F01

#### 3.3.1. Growth Kinetics and Production of Bacteriocin

**Figure 3:**
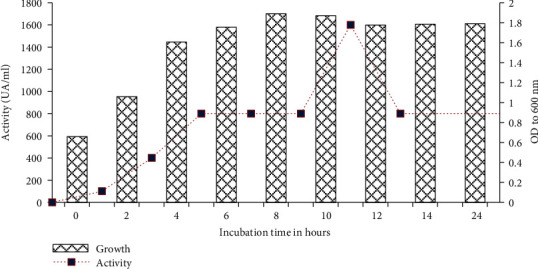
Growth and bacteriocin production kinetics of the strain *Lactococcus lactis* FO1 at 37°C.

The kinetics of growth and production of bacteriocin by strain F01 is illustrated in Figure 3. It was observed that the production of bacteriocin starts from the tenth hour of incubation and the maximum activity was observed in the middle of the stationary phase 12 h. Also, a decrease in bacteriocin activity was observed after 14 h of incubation and an increase at the twenty-fourth hour of incubation, but this activity remains however lower than that observed after 12 h of incubation. This result simply shows that the production of bacteriocin can therefore be correlated to the production of biomass (quorum sensing mechanism). Similarly, the decrease in activity can simply be explained by a decrease of the nutrients in the growth medium and by the presence of proteolytic enzymes [35] in the medium. The new peak of antibacterial activity production can be explained by the synthesis of another antimicrobial molecule as well. Similar results have been reported by [36, 37]. To determine its spectrum of action, it was necessary to purify the bacteriocin produced by strain F01.

#### 3.3.2. Partial Purification of Bacteriocin (C4B)

**Figure 4:**
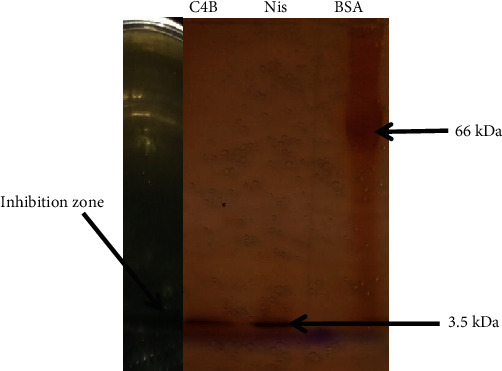
Molecular weight determination of bacteriocin by SDS PAGE and zymography of C4B. C4B: bacteriocin; Nis: nisin; BSA: bovine serum albumin.

Table 2 presents the results of the partial purification (protein amount, yield, specific activity, purification factor, and total activity) according to the different steps of the purification. An increase in protein concentration from 25.7 *μ*g/ml in CFS to 86.8 *μ*g/ml in PSA and from the latter value to 169 *μ*g/ml in PCM was observed. This increase was also observed at the level of the specific antimicrobial activity which was increased from 31.12 AU/*μ*g in the CFS to 37.82 AU/*μ*g in the PSA and to 37.86 AU/*μ*g in the PCM, whose purification yield was 40%. These results can be explained by the decrease of impurities after precipitation, hence the increase of the activities. According to the SDS-PAGE profile presented (Figure 4), bacteriocin peptide C4B from strain F01 has a molecular mass of around 3.5 kDa. The same observations were obtained by [24, 38].

#### 3.3.3. Action Spectrum of Bacteriocin (C4B)

The spectrum of C4B was determined against indicator strains and isolates (lactic and pathogenic bacteria from fresh fish and fish products), and the results obtained are presented in Table 3. We can deduce that NCFS was able to inhibit the growth of the lactic acid bacteria tested and wide range of pathogenic and nonpathogenic bacteria that were tested with different size of zone of inhibition. This broad inhibitory activity can be explained that C4B is a new bacteriocin with different structure and mode of action, different from the known class II bacteriocins synthesized by *Lactococcus* sp. Since bacteriocin C4B exhibited antimicrobial activity against both Gram-positive and Gram-negative bacteria, these results are in contradiction with those of [39], which state that the activity of bacteriocins produced by lactic acid bacteria are only directed towards Gram-positive bacteria. However, many studies are similar but with lower spectra than those obtained in this work such as the works of [40–42], which may indicate that the strain F01 produces a few bacteriocins with a different spectrum of activity. Indeed, given its spectrum of action, we wondered about its mode of action and its effect on certain resistance mechanisms (biofilm).

#### 3.3.4. Mode of Action of C4B

**Figure 5:**
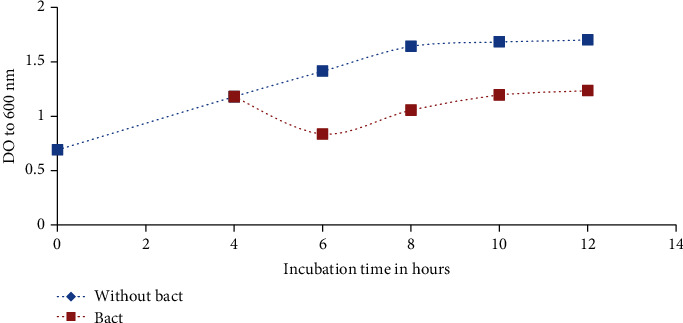
Effect of C4B on the growth of *Salmonella* Typhimurium ATCC 14028. Bact: bacteriocin.

**Figure 6:**
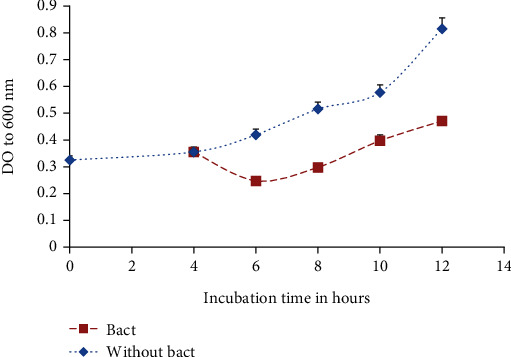
Effect of C4B on the growth of *Staphylococcus aureus.* Bact: bacteriocin.

The mode of action of C4B on the growth of *Salmonella* Typhimurium ATCC 14028 and *Staphylococcus aureus*, respectively, is illustrated in Figures 5 and 6. The addition of bacteriocin after 4 h of incubation results in a rapid decrease of the OD compared to the control (without bacteriocin) which were incubated under the same conditions. Then, we note a rebound resulting in a slight increase of the OD, but this value remains lower than that of the control both in the case of S*almonella* Typhimurium ATCC 14028 or *Staphylococcus aureus*. According to these results, C4B has a bactericidal effect against *Salmonella* Typhimurium ATCC 14028 and *Staphylococcus aureus*. The decrease of pathogen load is due to the bactericidal action of bacteriocin. Because according to its structure and class, it binds to the membrane of a bacterium through the disaccharide-pyrophosphate of lipid II and from there either interrupts the transport or the synthesis of peptidoglycan, which is a constituent of the membrane or creates pores in the membrane, which would lead to a disturbance of the acid-base balance in the cytoplasm and would directly cause the death of the cell. However, the slight increase of the load 2 h after the introduction of the bacteriocin is due to the decrease of the bacteriocin concentration in the medium; this decrease can be linked to the action of the proteolytic enzymes synthesized by the strain or bacteriocin titration. Similar results have been obtained by [43–45].

#### 3.3.5. Quantification of Biofilm Formation

**Figure 7:**
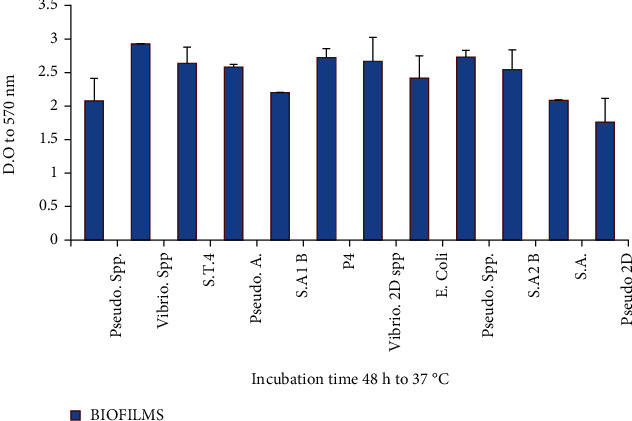
Quantification of biofilm formation.

Some isolates and strains were tested for their ability to form biofilms which are illustrated in Figure 7. Thus, strains of *Vibrio* sp. and *Pseudomonas* sp., respectively, showed a strong ability to form biofilms. However, strains of the *Staphylococcus* sp. genera also showed the ability to form biofilms but were lesser biofilm producers compared to the *Vibrio* sp. and *Pseudomonas* sp. The great capacity of *Vibrio* sp. and *Pseudomonas* sp. to form a high quantity of biofilms can be related to their capacity to more quickly metabolize the components of the medium making their latency timeless than the speed of division of the cells and surface. Similar results have been reported by [46, 47].

#### 3.3.6. Inhibition of Biofilm Formation

**Figure 8:**
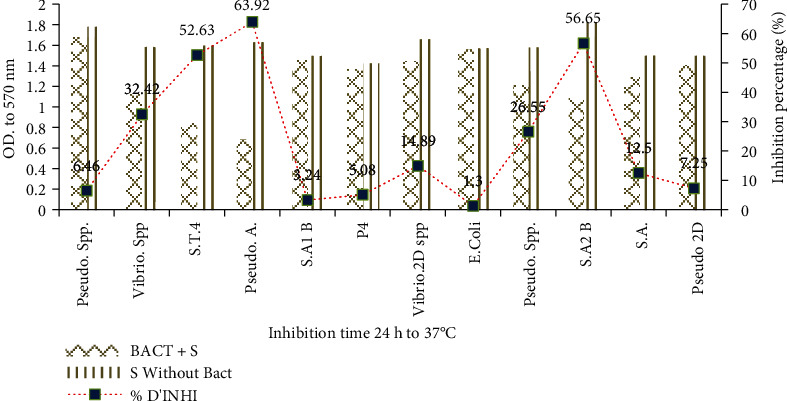
Inhibition of biofilm formation in percentages. Bact+S: bacteriocin+strain; S without Bact: strain without bacteriocin; % INHI: percent of inhibition.

The inhibitory capacity of biofilm formation by bacteriocin is illustrated in Figure 8. It can be highlighted that the percentage of inhibition depends in general on the isolate or the strain. Indeed, the *Escherichia* sp. strain remains the least inhibited with a percentage of inhibition of biofilm formation of 1.3 ± 0.003% followed by the *Staphylococcus* sp. 1B. isolate whose biofilms are also the least inhibited with a percentage of 3.24 ± 0.004%. However, *Pseudomonas aeruginosa* ATCC 15692 remains the most sensitive strain with a percentage of 63.92 ± 0.007% of biofilm inhibition. These results can be explained by the fact that the bacteriocin does not have a strong ability to penetrate the exopolymer layer contributing to the strong adhesion of cells to each other on one side and to the surface on the other side. However, the strong inhibition of *Pseudomonas* sp. and *Vibrio* sp. may be related to the speed of biofilm formation and dispersion. This result is in agreement with [48–50]. However, for later use, it was necessary to determine the effect of certain physicochemical parameters and chemical compounds on the bacteriocin.

#### 3.3.7. Effects of Temperature, pH, Enzymes, and Chemical Compounds on C4B

The effect of temperature, pH, enzymes, and chemical compounds on the C4B bacteriocin activity is summarized in Tables 4 and 5. It was found that the bacteriocin C4B is heat resistant, with 40% of the activity retained after 20 min of autoclaving. Bacteriocin also showed high pH stability, but lost its activity above pH 10. Also, bacteriocin C4B lost activity after treatment with different enzymes. A particular resistance pattern was observed after treatment with organic solvents; chemical compounds such as SDS, Tween 80, Tween 20, and urea; and a high concentration of NaCl. These characteristics of bacteriocin make it very suitable for use as a preservative in the food industry because it is extremely stable to changes in temperature, pH, and chemicals. Similar results were obtained for bacteriocins isolated from natural isolates belonging to class I and class II [38, 51, 52].

### 3.4. Determination of MIC for C4B

The determination of MIC for C4B and comparison with those of different antibiotics on strains most of which are recognized as multiresistant according to WHO in 2017 were carried out during this work, and the results were presented in Table 6. The MIC values of *Escherichia coli* strain were 30 or even 40 *μ*g/ml for some of the antibiotics, while MIC for the bacteriocin C4B was 21.7 *μ*g/ml. For the *Salmonella* Typhimurium, MIC varied from 3.25 ≤ MIC > 35 *μ*g/ml, while for bacteriocin C4B was 21.7 *μ*g/ml and that of *S. aureus* varied from 0.507 ≤ MIC ≥ 30 *μ*g/ml, while for bacteriocin C4B was 10.85 *μ*g/ml. Most probably, the inhibition capacity of C4B bacteriocin is linked to its mechanism of action, which is more specific than those of antibiotics. Similar results were reported by [53, 54]. However, for its probable future use as an additive, it was necessary to perform a toxicity test (hemolytic test).

### 3.5. Determination of the Hemolytic Activity of C4B

Table 7 shows the hemolytic activity of C4B according to different concentrations. It can be concluded that the hemolytic activity is dependent on the concentration because at the C4B concentration of 86.8 *μ*g/ml, the percentage of hemolysis is 21.48 ± 0.0043%, while when this concentration is half of that 43.4 *μ*g/ml, it is observed that the hemolytic activity is reduced to 2.47 ± 0.0038%. However, at a concentration of 10 *μ*g/ml, the percentage of hemolysis is almost zero of which 0.7 ± 0.0029% is almost equal to that of nisin at the same concentration. These results are in agreement with those reported by [32, 55].

### 3.6. Association of Bacteriocins with the Cell Wall of the Producing Strain

Indeed, to optimize the yield of the bacteriocin, it was important to know the factors (association of the bacteriocin with the cell wall of the producing strain) which can influence its yield. The association of bacteriocin with the cell wall was evaluated, and the results are presented in Table 8. It shows that 3.86 ± 0.00386 *μ*g/ml of protein was obtained after desorption at pH 1.5 and 6.49 ± 0.0013 *μ*g/ml at pH 2. The results obtained are explained by the capacity of the proteins to form interactions with the membrane receptors at a certain pH close to 6. The latter is made up of amino acid molecules which are made up of amine and acid groups that give them a charge, and this charge varies according to the pH of the medium as well as their activities. Similar results were obtained in the case of the work of [44, 56, 57].

### 3.7. Microbial Counts

**Figure 9:**
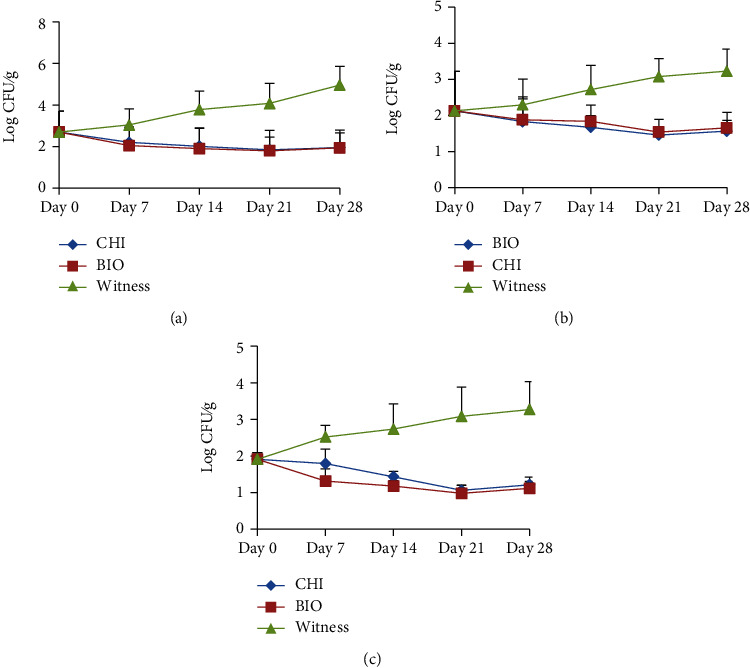
Evolution of bacterial load. (a) Evolution of the FAMT load; (b) evolution of the *Staphylococcus* sp. load; (c) Evolution of the *Vibrio* sp. load.

A load of total mesophilic aerobic flora (FAMT), *Staphylococcus* sp., *Vibrio* sp., *Salmonella* sp., and *Escherichia* sp. was monitored in the different fish sausage samples kept at 8°C for 28 days, and the corresponding results are presented in Figures 9(a)–9(c). It is shown that from day zero to day 21, the FAMT load varied from 2.70 ± 1.0 Log_10_ CFU/g to 1.79 ± 0.99 Log_10_ CFU/g for samples preserved with bacteriocin and from 2.70 ± 1.0 Log_10_ CFU/g to 1.83 ± 0.63 Log_10_ CFU/g for samples preserved with sodium benzoate. Similarly, the load of the control (sausages without preservatives) increases from 2.70 ± 1.40 Log_10_ CFU/g to 4.93 ± 0.90 Log_10_ CFU/g during the 28 days. However, samples with bacteriocin as a preservative did not differ significantly (*p* < 0.05) from samples with sodium benzoate as a preservative.

Regarding the *Staphylococcus* sp. load of the different samples, it varies from 2.14 ± 1.10 Log_10_ CFU/g to 1.45 ± 0.23 Log_10_ CFU/g for the samples with bacteriocin as a preservative and from 2.14 ± 1.10 Log_10_ CFU/g to 1.53 ± 0.35 Log_10_ CFU/g for those with sodium benzoate as a preservative between day zero and day 21. However, in the samples without preservatives, the load evolved from 2.14 ± 1.10 Log_10_ CFU/g to 3.26 ± 0.61 Log_10_ CFU/g during the 28 days. Analyses were also carried out on *Vibrio* sp. load; it was found that it varied from 1.91 ± 0.18 Log_10_ CFU/g to 0.97 ± 0.14 Log_10_ CFU/g for samples with bacteriocin as a preservative and from 1.91 ± 0.18 Log_10_ CFU/g to 1.03 ± 0.23 Log_10_ CFU/g for samples with sodium benzoate as a preservative. However, for the samples without preservatives, the lead fluctuated between 1.91 ± 0.18 Log_10_ CFU/g and 3.29 ± 0.76 Log_10_ CFU/g during the 28 days of storage at 8°C. However, it was observed that the *Salmonella* sp. and *Escherichia* sp. load was zero from day zero. Thus, it was observed that the samples with bacteriocin as a preservative preserved the sausages better concerning the bacterial load obtained after microbiological analysis. These results are explained by the bactericidal effect of the preservatives/bacteriocin on the one hand and also by the presence of spices that have an antimicrobial potential on the other hand. Thus, samples with bacteriocin as a preservative did not differ significantly (*p* > 0.05) from samples with sodium benzoate as a preservative. However, the bactericidal effect of bacteriocin is the most important because the sausages with bacteriocin as a preservative have a low load compared to those with sodium benzoate. The evolution of the bacterial load after day 21 can also be explained by the increase in water content and pH which can have an impact on the activity of bacteriocin and even sodium benzoate. In addition, other factors intrinsic to the food may inhibit or reduce the activity of bacteriocin or sodium benzoate because bacteriocin interacts with certain food additives or ingredients. In general, it is observed that the load of the different pathogenic bacteria evaluated is lower than the standard during the 28 days of storage at 8°C as it is the case of *Vibrio* sp. obtained which is significantly lower (*p* < 0.05) than that recommended for *Vibrio parahaemolyticus* in the USA (100,000 CFU/g). Work along these lines has been reported by [58–60].

## 4. Conclusion

The aim of this study was to screen bacteriocin-producing LAB from fresh fish and fish products, select a prominent strain, characterize the bacteriocin produced, and evaluate its potential to be used as biopreservatives. Our results demonstrate that *Cyprinus carpio* fish gut was weakly colonized by LAB and that bacteriocin produced by the identified endogenous LAB strain has strong ability to inhibit the growth of host-derived and non-host-derived pathogens. In addition, bacteriocin exhibits very good thermal and pH stability, and according to the hemolytic test, it is nontoxic for eukaryotic cells and was able to reduce the bacterial load in fish sausage during storage. Therefore, it could be considered as a reliable and inexpensive natural preservative in the biopreservation of fish sausages or seafood, especially in tropical countries of sub-Saharan Africa, or as an additional strategy to antibiotics.

## Figures and Tables

**Table 1:** Phenotypic identification of the bacteriocin-producing strain.

Strain	*Lactococcus lactis* FO1			
Temperature	8°C	10°C	45°C	
Time	4 days	4 days	24 H	
Growth	+	+	+	
Ability to grow at alkaline pH (pH 9.6)				
Growth	—			
Ability to believe at different NaCl concentration				
Concentration	4%	6.5%	10%	
Growth	+	—	—	
Biogenic amine production	Lysine	Histidine	Phenylalanine	Arginine
	—	—	—	—

**Table 2:** Evolution of activity and protein concentration following purification stages.

Purification stage	Volume (ml)	Activity (AU/ml)	Total activity (AU/ml)	Protein (*μ*g/ml)	Specific activity (AU/*μ*g)	Purification factor	Yield (%)
CFS	200	800	160000	25,7	31,12	1	100
PSA	20	3200	64000	86,8	37,82	1,18	40
PCM	10	6400	64000	169	37,86	1,08	40

CFS: crude culture supernatant; PSA: ammonium sulfate precipitate; PCM: chloroform-methanol precipitates.

**Table 3:** Antimicrobial spectrum of C4B.

Indicator strains	Sources	Culture condition	Activity of C4B	
			NCFS	PSA
Lactic acid bacteria				
*Lb* sp. 2B.	Laboratory isolate	MRS. 37°C	+	+
*Lentilactobacillus senioris*	Strain	MRS. 37°C	+++	+++
*Lb* sp. M6p.	Laboratory isolate	MRS. 37°C	+++	+++
*Lb* sp. Til2MB.	Laboratory isolate	MRS. 37°C	+++	+++
Pathogenic Gram+ bacteria				
*Staphylococcus* sp.	Clinical strain	BHI. 37°C	++	+++
*Bacillus subtilis*	ATCC13453	BHI. 37°C	++	++
*Bacillus* sp.	Clinical strain	BHI. 37°C	++	++
*Staphylococcus* sp. 2B.	Laboratory isolate	BHI. 37°C	++	+++
*Staphylococcus* sp. 1B.	Laboratory isolate	BHI. 37°C	++	++
*Clostridium* sp.	Clinical isolate	BHI. 37°C	++	++++
Pathogenic Gram− bacteria				
*Salmonella* Typhimurium	ATCC 14028	BHI. 37°C	++	+++
*Pseudomonas aeruginosa*	ATCC 15692	BHI. 37°C	++	+++
*Klebsiella pneumonae*	ATCC 35595	BHI. 37°C	++	+++
*Pseudomonas* sp.	Clinical isolate	BHI. 37°C	++	++++
*Pseudomonas* sp.	Clinical isolate	BHI. 37°C	++	+++
*Escherichia* (SMR) sp.	Clinical isolate	BHI. 37°C	++	++
*Shigella* sp.	Clinical isolate	BHI. 37°C	++	+++
*Escherichia* sp. 2B.	Laboratory isolate	BHI. 37°C	++	+++
*Escherichia* sp. 3B.	Laboratory isolate	BHI. 37°C	++	+++
*Serratia* sp.	Clinical isolate	BHI. 37°C	—	—
*Yessinia* sp.	Clinical isolate	BHI. 37°C	++	++++
*Salmonella* sp. 3B.	Laboratory isolate	BHI. 37°C	++	+++
*Vibrio* sp. 2DS.	Laboratory isolate	BHI. 37°C	++	+++
*Vibrio* sp. 1DS.	Laboratory isolate	BHI. 37°C	++	+++
*Salmonella* sp. 2B.	Laboratory isolate	BHI. 37°C	++	+++
*Pseudomonas* sp. Z.	Clinical isolate	BHI. 37°C	++	+++

PSA: ammonium sulfate precipitate; NCFS: neutralized cell-free supernatant;–: no inhibition; +: 1.0–3.0 mm (weak); ++: 3.1–6.0 mm (good); +++: 6.1–14.0 mm (very good); ++++: >14.0 mm (strong).

**Table 4:** Effect of heat treatment, pH, and enzymes on bacteriocin activity.

	Activity (mm)	Activity (AU/ml)	Residual activity (%)
Temperature			
Control	10	800	100
80°C 10 min	10	800	100
100°C 30 min	10	800	100
100°C 60 min	8	400	80
100°C 90 min	6	400	60
100°C 120 min	6	400	60
121°C 2 bar 20 min	4	200	40
Effect of pH			
Control	10	800	100
pH 2	10	800	100
pH 3	10	800	100
pH 4	10	800	100
pH 5	10	800	100
pH 6	10	800	100
pH 7	10	800	100
pH 8	10	800	100
pH 9	10	800	100
pH 10	10	800	100
Effect of enzymes			
Trypsin		**—**	
Proteinase K		**—**	
Pepsin		**—**	

The control is an untreated C4B bacteriocin solution. Residual activity is the percentage of activity in mm after treatment over the activity in mm of the control.

**Table 5:** Effect of organic solvents, detergents, and surfactants on bacteriocin activity.

	Activity (mm)	Activity (UA/ml)	Residual activity (%)
Effect of organic solvents			
Control	8	800	100
Acetone	8	800	100
Chloroform	8	800	100
Methanol	8	800	100
Ethanol	8	800	100
Effect of detergents and surfactants			
Control	10	800	100
SDS	7	400	70
Urée	10	800	100
Tween 20	10	800	100
Tween 80	10	800	100
Triton X-100	10	800	100
Effect of EDTA			
Control	8	800	100
1%	10	800	125
2%	13	1600	162.5
3%	14	1600	175
4%	14	1600	175
5%	16	3200	200
Effect of NaCl			
Control	8	800	100
1%	10	800	125
2%	10	800	125
3%	11	800	137.5
4%	12	1600	150
5%	12	1600	150
6%	13	1600	162.5
7%	13	1600	162.5

The positive control consists of an untreated solution of bacteriocin C4B. Residual activity is the percentage of activity in mm after treatment over the activity in mm of the control.

**Table 6:** MIC of antibiotics and bacteriocin.

Strain	MIC value in *μ*g/ml									
	Bact	Aug	Cipro	Tetra	Imi	Vanco	Cefo	Peni	Levo	Doxo
*E. coli*	21.7	>37.5	>32.5	>40	>32.5	>35	15	>30	>30	>35
*S.* Typhi	21.7	18.75	8.125	40	3.25	8.75	3.75	15	30	>35
*P. aeruginosa*	21.7	37.5	0.253<	5	16.25	35	>30	>30	1.875	4.375
*S. aureus*	10.85	18.75	0.507	2.5	16.25	35	>30	15	0. 937	1.094
*Vibrio* sp.	10.85	18.75	0.253<	20	8.125	2.187	15	30	0.234<	4.375

Bact: bacteriocin; Aug: augmentin; Cipro: ciprofloxacin; Tetra: tetracycline; Imi: imipenem, Vanco: vancomycin; Cefo: cefoxitin; Peni: penicillin; Levo: levofloxacin; Doxo: doxycycline. *E. coli*: *Escherichia coli*; *S. Typhi*: *Salmonella* Typhimurium ATCC 14028; *Pseudo aeruginosa*: *Pseudomonas aeruginosa* ATCC15692; *S. aureus*: *Staphylococcus aureus* ATCC 35395; *Vibrio*: *Vibrio* sp.

**Table 7:** Percentage of C4B hemolytic as a function of concentration.

Bacteriocin	Concentration (*μ*g/ml)	Percentage of hemolysis (%)
C4B	86.8	21.48 ± 0.004
	43.4	2.47 ± 0.003
	10	0.7 ± 0.002
	2.5	0.2 ± 0.001
Nisin	10	0.6 ± 0.002
Triton X-100	0.5%	100

C4B: bacteriocin.

**Table 8:** Amount of protein as a function of desorption pH.

pH	Volume (ml)/mass (mg)	Protein (*μ*g/ml)	Total protein (*μ*g/ml)	Yield (%)	Loss (*μ*g/ml)	Loss as a function of extraction pH (*μ*g/ml)
/	20 (CFS)	13.54 ± 0.001	17.4 ± 0.590	/	/	2.63 ± 0.940
1.5	Cel (Ex-M)	3.86 ± 0.003		22.180	3.86 ± 0.003	
/	20 (CFS)	13.1 ± 0.001	19.64 ± 0.080	/	/	
2	Cel (Ex-M)	6.49 ± 0.001		33.040	6.49 ± 0.001	

Cel: cell; Ex-M: methanol extraction; CFS: crude culture supernatant.

## Data Availability

These data are available at the central library of the University of Dschang and at the Research Unit of Biochemistry of Medicinal Plants, Food Science, and Nutrition.
